# Surgical or non-surgical treatment of plantar fasciopathy (SOFT): study protocol for a randomized controlled trial

**DOI:** 10.1186/s13063-022-06785-w

**Published:** 2022-10-04

**Authors:** Stefan Møller, Henrik Riel, Jens Wester, Ane Simony, Bjarke Viberg, Carsten Jensen

**Affiliations:** 1 Department of Orthopaedic Surgery and Traumatology, Lillebaelt Hospital - University Hospital of Southern Denmark, Sygehusvej 24, 6000 Kolding, Denmark; 2 Center for General Practice at Aalborg University, Fyrkildevej 7, 9220 Aalborg, Denmark; 3 Department of Health Science and Technology, Faculty of Medicine, Aalborg University, Aalborg, Denmark; 4 Department of Physiotherapy, University College of Northern Denmark, Aalborg, Denmark

**Keywords:** Plantar fasciopathy, Heavy-slow resistance training, Radiofrequency microtenotomy, Foot Health Status Questionnaire

## Abstract

**Background:**

Plantar fasciopathy is the most common reason for complaints of plantar heel pain and one of the most prevalent musculoskeletal conditions with a reported lifetime incidence of 10%. The condition is normally considered self-limiting with persistent symptoms that often last for several months or years. Multiple treatments are available, but no single treatment appears superior to the others. Heavy-slow resistance training and radiofrequency microtenotomy for the treatment of plantar fasciopathy have shown potentially positive effects on short- and long-term outcomes (> 3 months). However, the effect of heavy-slow resistance training compared with a radiofrequency microtenotomy treatment is currently unknown. This trial compares the efficacy of heavy-slow resistance training and radiofrequency microtenotomy treatment with supplemental standardized patient education and heel inserts in improving the Foot Health Status Questionnaire pain score after 6 months in patients with plantar fasciopathy.

**Methods:**

In this randomized superiority trial, we will recruit 70 patients with ultrasound-confirmed plantar fasciopathy and randomly allocate them to one of two groups: (1) heavy-slow resistance training, patient education and a heel insert (*n* = 35), and (2) radiofrequency microtenotomy treatment, patient education and a heel insert (*n* = 35). All participants will be followed for 1 year, with the 6-month follow-up considered the primary endpoint. The primary outcome is the Foot Health Status Questionnaire pain domain score. Secondary outcomes include the remaining three domains of the Foot Health Status Questionnaire, a Global Perceived Effect scale, the physical activity level, and Patient Acceptable Symptom State, which is the point at which participants feel no further need for treatment.

**Discussion:**

By comparing the two treatment options, we should be able to answer if radiofrequency microtenotomy compared with heavy-slow resistance training is superior in patients with plantar fasciopathy.

**Trial registration:**

ClinicalTrials.gov NCT03854682. Prospectively registered on February 26, 2019.

**Supplementary Information:**

The online version contains supplementary material available at 10.1186/s13063-022-06785-w.

## Background

Plantar fasciopathy (PF), formerly labelled as “plantar fasciitis”, is the most common reason for complaints of plantar heel pain and one of the most prevalent musculoskeletal conditions with a reported lifetime incidence of 10% and period prevalence of 3.6–7.0% [1–4].

People with PF report pain during the first steps in the morning or after inactivity which improves with ambulation and worsens during the day [3, 5, 6]. Patients with PF are also prone to greater levels of depression, stress, anxiety, and experience limitations in both mobility and health-related quality of life compared with pain-free individuals [7, 8].

PF has historically been considered a self-limiting condition where 60–80% are expected to achieve symptom-free status within 12 months with conservative treatments [9–11]. This view of PF as a self-limiting condition has been challenged by research [9, 12]. According to a recent cohort study, approximately half of patients referred to a specialized secondary care clinic still experienced pain 10 years after treatment [12]. Furthermore, 40% of patients in a randomized controlled trial still had symptoms 2 years after a treatment protocol consisting of plantar fascia-specific stretching and wearing insoles [9].

### Non-surgical versus surgical treatment

Some of the challenges in achieving a satisfactory recovery after being diagnosed with PF is finding a suitable treatment protocol, where the quality of evidence has been reported to be mostly low or moderate for many of the interventions [13, 14]. The treatment is also reported to be characterized by inconsistent management of the disorder [13–15]. This inconsistency could partly be due to the fact that no single modality treatment seems to be superior to others [14, 16]. According to a recent systematic review by Babatunde et al., none of the investigated treatments (i.e. corticosteroid injection, non-steroidal anti-inflammatory drugs, therapeutic exercise, orthoses, and/or extracorporeal shockwave therapy) was superior to the others [16].

Heavy-slow resistance training (HSRT) was not included in the above-mentioned systematic review, but it is generally known for significant efficacy in the rehabilitation of lower-limb tendinopathies [17–20]. Rathleff et al. reported a greater reduction in self-reported pain after 3 months of plantar-specific strength training and insoles compared with traditional treatment with plantar-specific stretching and insoles for PF patients [20]. HRST has also shown better micro-structural improvements in tendon tissue and pain reduction compared to traditional eccentric training of the patella and Achilles tendon [17, 18].

HSRT, as a form of treatment for all types of chronic injuries, has potentially gained ground because the training is easy to perform for the patient and has shown good clinical applicability for the healthcare staff. This is despite the fact that the effects of HSRT for patients with PF have only been compared to stretching, and the only other research in the area has compared different exercise protocols of the same exercise [20, 21].

Traditionally, surgical treatment for PF is considered as a last resort but could alternatively be considered as a treatment option between 6 and 12 months from symptom onset [22–24]. A newer minimally invasive treatment modality known as radiofrequency microtenotomy (RFM) for the treatment of PF has shown promising results in short- and long-term results. The treatment consists of a probe which is passed down through the skin to the tendon where radio waves are discharged into the tendon’s affected area through independent inserts. This excites and heats the soft tissue to relatively low temperatures (40–70 °C). The heating and the radio waves are believed to create a renewed inflammatory response, as well as positively affect the local tissue’s growth factors and nociceptors [25, 26]. The treatment is gentle, and several non-randomized studies have shown good results and minimal complications in the treatment [25–29]. RFM treatment is reportedly as effective as the more extensive and invasive surgery, open plantar fasciotomy. Moreover, a faster return to normal activities, fewer complications, and reduced pain are seen with RFM treatment compared to open plantar fasciotomy [28–32].

To date, RFM has not yet been compared to other types of conservative treatments including HRST. By comparing HSRT with RFM treatment, we should be able to answer if RFM is superior to performing exercises.

## Objectives and hypothesis

The purpose of this trial is to investigate the efficacy of HRST versus RFM by comparing patients’ pain levels with the Danish version of the Foot Health Status Questionnaire (FHSQ-DK) 6 months after treatment initiation. Both groups will in addition receive standardized patient education and a heel insert. Our hypothesis is that surgical treatment is better than non-surgical treatment measured on the FHSQ-DK (pain) score after 6 months.

## Methods

### Design

A prospective randomized clinical superiority trial is performed with a balanced randomization (1,1). Reporting of the protocol follows the Standard Protocol Items: Recommendations for Interventional Trials (SPIRIT) statement [33] (checklist uploaded as Additional file 1). Before the inclusion of the first participant, the trial was registered at ClinicalTrials.gov (NCT03854682).

### Participants and recruitment

Individuals referred to the Department of Orthopaedic Surgery and Traumatology, Kolding, Lillebaelt Hospital in Denmark, with plantar heel pain will be examined and screened for potential participation. The recruitment will be performed in a standard outpatient clinic setting by a specially trained healthcare personnel (SM), who has a special interest in PF pathology. The recruiting healthcare personnel is also trained and experienced in ultrasound scanning of the plantar fascia thickness with regard to the diagnostic evaluation.

Participants will attend the baseline, and a link to the questionnaires used will be sent via REDCap (Vanderbilt University, Nashville, TN, USA) to participants’ e-mail addresses for the 4-week, 12-week, 26-week, and 52-week follow-ups.

### Eligibility criteria

The diagnosis of PF is based on the diagnostic criteria according to the literature [5, 6, 21]: pain must be well-defined with the initiation of pain after rest (first-step pain), and the gait pattern should be changed to relieve the foot. Palpation is performed to identify the area of pain, which should be located at the proximal plantar fascia insertion area, and the patient is examined for differential diagnoses (e.g. Tinel’s sign over the tarsal tunnel, fat pad syndrome, anamnesis of bilateral debut of symptoms). Findings are verified with ultrasound, where a plantar fascia thickness of > 4 mm should be present.

Patients diagnosed with PF, who are older than 18 years; have well-defined plantar heel pain, a pain intensity of 30/100 within the last 7 days (VAS), and palpation tenderness at the plantar fascia insertion; and experience initiation pain after rest (first-step pain), are offered participation in the study. The duration of symptoms must be over 9 months. People with systemic diseases, diabetes, previous heel surgery, and Tinel’s signs over the Tarsal tunnel; pregnant women; and people who have received medical or physiotherapy treatment/cortisone injection within the last 3 months or have a fascia thickening (< 4 mm) are excluded (see Table 1 for the in- and exclusion criteria).

**Table 1:** Inclusion and exclusion criteria

Inclusion	Exclusion
≥ 18 years	History of systemic diseases and neuropathy
Well-defined plantar heel pain	Diabetes
VAS ≥ 30/100 within the last 7 days	Pregnant
Palpation tenderness at the plantar fascia insertion	Previous heel surgery
First-step pain	Tinel’s signs over the tarsal tunnel
Duration of symptoms ≥ 9 months	Received medical/physical therapy treatment and/or cortisone injection within the last 3 months
	Fascia thickening (< 4 mm)

Patients are informed orally and in writing, and if they are willing to participate, they will sign a declaration of consent before inclusion. After this, randomization will take place. Patients who want a consideration period will be given a statement of consent and “subjects’ rights” and will be contacted by telephone after 3 days.

All patients are informed about the latest knowledge within the area of PF, including the expected prognosis, as well as risks. They will also receive information about the trial’s content, purpose, and time horizon, including a right to withdraw their consent at any time.

It is permitted that patients who have performed self-administered treatment in the form of foot massage, thermotherapy, and stretching of the fascia may continue with this, provided this has been done for a minimum of 4 weeks before inclusion.

### Randomization

Shortly after the written consent is signed, the patient will be contacted by a research secretary for further allocation. The randomization is performed by a research secretary with a software program (REDCap), which generates the allocation sequence in blocks of 10 patients, where 5 will be randomized to HRST treatment and 5 will be randomized to RFM treatment. The randomization within the blocks of 10 is random and is done without stratification. The randomization is done by the secretary in a separate room before contact.

### Blinding

The nature of the trial means that the group allocation cannot be hidden (blinded) from the patient or the researchers involved. To reduce the risk of bias, the assessor and statistician are blinded, and patients are encouraged not to state their assigned treatment in case of contact.

### Sample size

Based on a standard deviation of 19.7 points, a two-sided significance level of 0.05, and a power of 80%, it will require 32 participants in each group to be able to detect a difference in the clinically relevant 14.1 points in the pain domain of FHSQ-DK [34]. To account for possible drop-outs, we will include a total of 70 participants.

### Pilot study

Prior to the study, a pilot study was made in which the patients’ preference (*n* = 24) for treatment type (due to patients’ equipoise) [35] was compared to symptom duration and previous treatment. Patients were presented with a short, standardized text and video of the two types of intervention and asked if they would choose the non-surgical or surgical treatment. No significant preference was found compared to the duration of symptoms or prior treatment.

### Statistical analyses

All statistical analyses have been planned in cooperation with a statistician assigned to the statistical department of OPEN (Odense, Denmark) that are responsible for data storage in REDCap. The primary analysis will investigate the between-group difference in FHSQ-DK pain. The analyses will be performed by a blinded data analyst (at the group level) using a linear mixed effect model with the participant as a random effect and time (baseline, 4, 12, 26 and 52 weeks) and group allocation (HRST or RFM) as fixed effects. The model will include the baseline measurements with the constraint of no group difference at baseline.

An intention-to-treat analysis is planned, where a per-protocol analysis could be carried out (as a supplementary analysis), provided that a very varying following is seen with the intervention procedures. Missing outcome data will be imputed using a multivariate model based on a normal distribution.

It is also planned that analyses of the mean values of the secondary continuous outcomes will be done by using linear mixed models. Resample bootstrap will be used in case there are substantial ambiguous data for these outcomes.

The time when PASS is obtained between the two groups is compared. If a participant changes PASS multiple times (e.g. achieving PASS before 12 weeks, reporting not to have achieved PASS at 26 weeks, and then having achieved PASS again at the 52-week follow-up), only time to the first PASS achieved is used in the analysis [36]. We will calculate the relative risk of achieving PASS at each follow-up.

In an additional analysis, we will by linear mixed models investigate if anthropometric baseline values, e.g. gender, age, and length of symptoms influence the primary outcome.

Dropouts will be registered, and the reasons for this will be noted.

### Interventions

#### General intervention

All participants are provided with a relieving heel insert or they can continue with their own preferred insert. This is permitted, since no difference in the effect of different inserts has been found [37].

Both groups will receive access to video-based patient education to support adherence to the recommendations during an intervention [38–41].

The possibility of informative control sessions is offered in each group. The training group meets for physical control of the exercise itself, and clarification of any relevant questions will be answered. The surgical group can call if guidance is needed. If they present any signs of complications, this will be handled by the on-call staff and registered and reported cf. regulations.

#### Surgical treatment

The treatment is performed percutaneously through plantar access with the patient under local anaesthesia. Pre- and postoperative information is given orally, and they have access to a video presentation with information and educational content (imparting knowledge of loading, pain, and adaptation) on the procedure and post-operative regime. The treatment is performed in an area of approx. 4 × 5 cm corresponding to the affected area of the fascia and injected with lidocaine and adrenaline. Twenty-five independent insertions are made through the skin with 2-mm k-wires. Then, a probe is inserted, and radiofrequency energy discharge is performed in 2 rounds, partly corresponding to the surface of the fascia plantaris (light contact of the tendon tissue with the tip of the probe) and subsequent perforation in the tendon substance itself. Water cooling is performed at 3 drops per second.

Compressive bandages are then applied. The patient is instructed in a sedentary to light activity regimen for the first 2 days without support on the foot, due to the risk of bleeding. Removal of the large dressing is done by the patient at home after 3–4 days, and the inner bandage can be removed after a week. The patient is guided in unloaded activity with venous pump exercises for the first 2 days, and then the patient must start with partial loading the following 3–4 days with increased load to full weight bearing after 1 week.

The time of return to work is divided into the degree of strain of standing and walking activity: light work and weight-bearing activities (approx. 7 days), moderate (approx. 14 days), and hard (approx. 4 weeks). The procedure is performed by an experienced chief physician in orthopaedic surgery, who has worked with RFM treatment since 2013.

#### Non-surgical treatment

The non-surgical treatment consists of a specific strength exercise (HSRT), according to the protocol of Rathleff et al., which consists of a one-legged heel raise with a rolled-up towel under the forefoot [20]. The exercise should activate the windlass effect and increase the mechanical stress on the tendon (fascia plantaris) [42]. The towel should ensure the maximum comfortable dorsiflexion of the toes. Patients are instructed to perform the exercise on a stair tread, a thick book, or similar, so that the heel during the exercise is lowered below the horizontal plane. To ensure maximum effort and avoid postural balance issues, the patient should secure support from, for example, a railing or wall structure. The exercise is performed every other day with as many sets as possible, and as heavy as possible, but not heavier than the patient can perform 8 repetitions per set.

The exercise is modified to be auto-regulated and not fixed, in the effort to improve patients’ adherence to the training [21]*.* Autoregulation refers to individuals self-selecting the exercise dosage (sets) based on their individual circumstances, response to exercise, and readiness to train [43].

The load is pro- and regressed from two- to one-leg ± backpack loading. The exercise is performed as 3 s/2 s/3 s concentric, isometric, and eccentric, respectively, followed by a 2-min break. The patient is encouraged to train for a minimum of 3 months and can stop his or her training 4 weeks after a satisfactory symptom state (patient acceptable symptom state) is achieved to emulate clinical practice. Pain during and after exercise is scored according to a numerical 0–10 scale in an assigned app (genoptræn.dk). Alternatively, a training diary in paper form can be handed out. Patients are instructed to reduce the load accordingly if the pain during or shortly after the exercise exceeds 5 out of 10.

### Outcomes/variables

Follow-up is planned at baseline and 1, 3, 6, and 12 months. The primary end-point is 6 months.

**Fig. 1:**
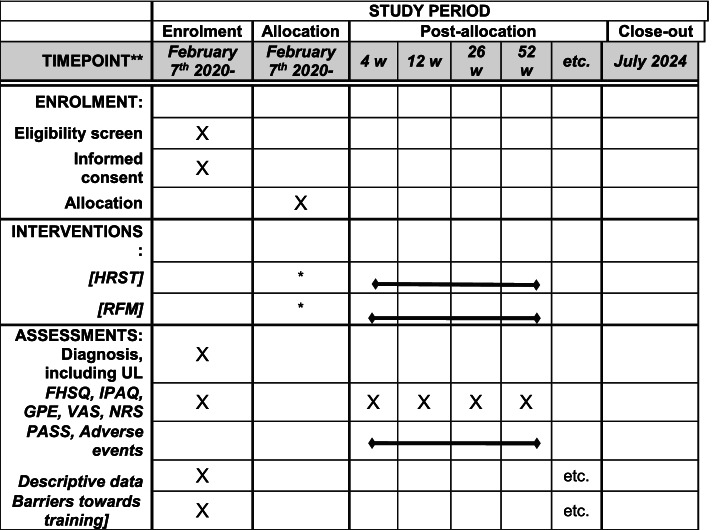
SPIRIT figure. Schedule of enrolment, interventions, and assessments. Asterisk symbol (*) indicates the following: treatment initiation as soon as possible after allocation

The assessment schedule is found in the SPIRIT figure (Fig. 1). During the screening, clinical examination, and follow-up, we will collect the following data (see below).

#### Primary outcome

The primary outcome is the mean pain domain score of the FHSQ-DK after 6 months [44].

FHSQ-DK consists of 13 questions that are divided into four domains: pain, function, footwear, and general foot health. It takes the patient less than 10 min to complete. FHSQ scores for each domain range from 0 (worst) to 100 (best). The minimal clinically important difference in the pain domain is reported to be 14.1 points, 7.4 points for function, and 9.2 points for footwear [34]. FHSQ-DK is a Danish validated translation of the original questionnaire [44]. FHSQ has been reported as the preferred questionnaire for patients with plantar fasciopathy [45].

The selected HRST regimen has shown a larger pain reduction after 3 months, but not after 12 months compared to a stretching group [20], and the HRST regimen may not be sufficient to achieve Patient Acceptable Symptom State in most people with plantar fasciopathy after 12 weeks [21]. It can take time to manifest changes to a training regimen, and therefore, we aimed for a primary end-point at 6 months.

#### Secondary outcomes

The following are the secondary outcomes:

1. The other domains of FHSQ-DK (function, footwear, and general foot health).
2. Numeric Rating Scale (NRS) for pain during and after training (genoptræn.dk).
3. Global Perceived Effect (GPE) scale: Overall satisfaction with result and treatment measured by using GPE which is a recommended responder criterion and is rated on a 7-point Likert scale. A subjective assessment of change is made for the areas: pain, symptoms, activity, and treatment [46, 47].
4. Physical Activity Questionnaire (IPAQ): Physical activity is measured by self-reported participation in sports, and leisure activities are measured by IPAQ. The questions relate to the time spent on physical activity during the last 7 days. The patient indicates the type of activities performed and divided into number of days or time in minutes/hours. Assessment is categorized into work, activities that form part of the work at home or in the garden, activities to get from one place to another, and leisure time activities related to relaxation, exercise, or sports [48, 49].
5. Patient Acceptable Symptom State (PASS): PASS is used as a measure of the time when the patient’s symptoms are acceptable and no further treatment is needed [50].

#### Ultrasound scan for diagnostic value

All patients will be examined with an ultrasound scan of the plantar fascia thickness at the insertion of the calcaneus. The positive predictive value for assessing an ultrasound scan is 0.83–0.90, while the negative predictive value is 0.57–0.89 in a group with symptoms [51–53]. To increase the reliability, an average of 3 measurements is made [54]. Here, a plantar fascia thickness above 4 mm. must be seen around the proximal insertion of the calcaneus [55].

#### Other outcome variables

Adherence is measured as the number of performed training sessions. “Very good” is achieved when at least 75% of the training is completed. “Good adherence” is achieved by completing 50–74%, “moderate” by 25–49%, and “bad” by less than 25%.

1. Adverse events
  - Number of sessions (% of total) that are completed with self-reported pain > 5 on the NRS 0–10 scale.
  - Number of sessions (% of total) that are not completed due to pain or challenges related to the affected leg/foot. The cause for not completing is shortly noted.

Adherence and pain are recorded as an immediate self-reported assessment after the exercise using the NRS scale. The pain score is grouped as 0–2 (safe), 3–5 (acceptable), and > 5 (high pain risk). Thus, the total percentage of the completed training sessions with acceptable pain can be assessed and evaluated in relation to plantar-specific adverse effects.

2. Barriers towards training

It is assessed on a Likert scale whether the patient experiences any barriers towards a training protocol before the start of the intervention and is compared with adherence towards the agreed-upon training sessions and primary outcome.

#### Anthropometric variables

Age, sex, weight, height, BMI, right/left side symptoms, symptom duration, number of sick days before and after the intervention, level of education, type of job (sedentary vs. active), consumption of painkillers, smoking, and co-morbidity are recorded. Self-administered treatment is registered. Complications and side effects are collected at follow-up or reported by the on-call staff in acute cases.

#### Ethics

The study complies with the Declaration of Helsinki, and the study is approved by the regional science ethics committee and the Danish Data Protection Agency before commencement. The potential risks and benefits of participation are presented to patients, and the necessary insurance conditions are ensured before start-up.

## Discussion

This is the first RCT, as far as we are aware, that investigates the effectiveness of HRST compared to RFM treatment for PF. While the condition is well-described and there are multiple treatment options available, no single treatment modality has shown superiority [14–16], and the quality of evidence has been reported to be mostly low or moderate for many of the interventions [13].

In general, HRST seems to have good clinical applicability, and the training can be performed at home, which is also manageable and acts as a resource-saving modality. HSRT is increasingly being used for the rehabilitation of PF, despite its effects having only been examined in a few studies [20, 21] and a new best practice guide does not recommend HRST as a treatment for PF [13].

As an alternative surgical procedure, RFM may be offered to people with persistent pain from the PF condition and whose symptoms have not resolved following a conservative treatment regime. However, to date, there are no systematic reviews of the effectiveness of the various surgical procedures for PF compared to non-surgical treatment since comparative RCTs of surgical vs. non-surgical treatment modalities for PF are sparse.

For decades, the only other surgical option for recalcitrant cases of PF was performing a release of the plantar fascia, with the endoscopic technique favoured over the open release [56, 57]. These traditional surgical treatments of PF that includes complete plantar fasciotomy or endoscopic plantar fasciotomy, among other techniques, often require postoperative non-weight bearing and immobilization and therefore run the risk of developing venous thromboembolic diseases [57]. Such techniques are also generally known to risk the development of scar tissue and adhesion, as well as nerve entrapment [58].

Multiple retrospective studies suggest that minimal invasive surgery performed with RFM may be a suitable option for PF if conservative treatment fails and seems to be a safe treatment with predominately high satisfaction rates (see Table 2), and in general, minimally invasive procedures seem to minimize skin healing problems, nerve injuries, infection, and prolonged recovery time, thereby allowing early return to normal activities [59]. The percutaneous approach does not require soft tissue dissection and retraction as compared with the open approach, so wound healing and recovery might be more rapid [58, 59]. As this procedure does not involve cutting of the plantar fascia, the risk of fascia rupture is also potentially minimized and patients are able to transition into normal shoe gear quicker and experience a more rapid return to daily activities with minimal loss of time from work [31, 58, 59].

**Table 2:** Reported complications and satisfaction with RF treatment

Author	Number	Complications reported	Not satisfied
Bagali et al. (2016) [59]	70	None	2.86%
Chou et al. (2016) [27]	48	None	7.3%
Hormozi et al. (2011) [31]	14	None	7.14%
Sean et al. (2010) [32]	14	None	14.3%
Shah et al. (2016) [25]	3	None	NR
Sorensen et al. (2011) [28]	21	*N* = 1^a^	9.52%
Tay et al. (2012) [26]	48	None	28.6–33.3%
Weil et al. (2008) [30]	10	None	10%

*NR* not reported

^a^Flexor hallucis longus tendinopathy

Since RFM seems to be a safe treatment with high satisfaction rates, it seems compelling to compare RFM treatment with the active treatment modality HRST, which has shown significant efficacy for tendinopathy in the lower extremity.

### Perspective

Patients ask for both short-term and long-term pain reduction, where RFM and HRST may potentially offer this based on different adaptive mechanisms and possibly different temporal profiles. Despite our hypothesis that RFM treatment will be superior at 6 months (FHSQ-DK pain), the effects of HSRT could potentially be similar, which potentially would have a clinical impact on future indications for RFM treatment. There are obvious differences between the chosen treatments in the required time, personnel, costs, and materials. The treatment offered in the HRST group requires the least resources, whereas the RFM group requires the most resources in material, personnel, and specialized education. However, this difference may be equalized by the potential savings on a societal level in terms of reduced sick leave or on a personal level in terms of health-related improvement and personal expenses. Any future implementation will also be dependent on the patients’ experiences and the final results of the study.

### Practical implications of the study

Patients diagnosed with PF have a high risk (> 40%) of developing long-term pain. There is still no documentation for the best treatment for this condition. Should RFM treatment prove to be superior to the training protocol, this could give implications for changing recommendations of treatment and may contribute to patients with PF receiving more optimal treatment. This could also help to support the opportunity to maintain employability, as well as mobilizing and keeping people more active with increased quality of life.

### Trial status

Recruitment was started on February 7, 2020, and the first participant was included on the same date. One amendment has been made to the local ethics committee and has been approved (version 3.5, March, 2022). The amendment concerns a change of the responsible person for the research project from being CJ to AS, a prolonged research period to 1.7. 2025, and a change in the randomization method performed in REDCap. When this protocol was submitted for publication, a total of 31/70 (54 screened) participants had been included in the trial. We expect recruitment to be completed by July 2024.

## Supplementary Information

**Additional file 1.** Standard Protocol Items: Recommendations for Interventional Trials (SPIRIT) statement.

## Data Availability

Data will be made available upon reasonable request.

## Abbreviations

FHSQ: Foot Health Status Questionnaire
HRST: Heavy-slow resistance training
PF: Plantar fasciopathy
RFM: Radiofrequency microtenotomy
SPIRIT: Standard Protocol Items: Recommendations for Interventional Trials
VAS: Visual analogue scale
NRS: Numeric Rating Scale
